# The angiotensin receptor and neprilysin inhibitor, LCZ696, in heart failure: A meta-analysis of randomized controlled trials

**DOI:** 10.1097/MD.0000000000030904

**Published:** 2022-10-14

**Authors:** Yan Chen, Qian He, Dun-Chang Mo, Long Chen, Jia-Lu Lu, Rui-Xing Li, Jie Huang

**Affiliations:** a Department of Geriatrics, Wuxiang Branch of Nanning Second People’s Hospital, Nanning, Guangxi, China; b Radiotherapy Department, Nanning Second People’s Hospital, Nanning, Guangxi, China; c ENT & HN Surgery Department, Nanning Second People’s Hospital, Nanning, Guangxi, China.

**Keywords:** ACEI/ARB, heart failure, LCZ696, meta-analysis, neuroendocrine inhibitor

## Abstract

LCZ696 is a novel neuroendocrine inhibitor that has been widely used in heart failure (HF). However, its advantage over other neuroendocrine inhibitors, such as angiotensin-converting enzyme inhibitors (ACEis) and angiotensin-receptor blockers (ARBs) has not been fully elucidated. This study aimed to provide the latest evidence regarding the efficacy and safety of LCZ696 as compared to other ACEis and ARBs with regards to the treatment of HF.

**Methods:**

We systematically searched databases, including PubMed, Embase, and the Cochrane Library, for relevant randomized controlled trials (RCTs). The outcome measures included all-cause mortality, rate of hospitalizations for HF, rate of death from cardiovascular causes, change in N-terminal pro-brain natriuretic peptide (NT-proBNP) levels, and decline of renal function.

**Results:**

Five RCTs involving 19,078 patients were identified. The meta-analysis indicated that LCZ696 was associated with a significant reduction in all-cause mortality (hazard ratio [HR] = 0.84; 95% confidence interval [CI], 0.76–0.93; *P* = .0005), rate of hospitalizations for HF (HR = 0.80; 95% CI, 0.73–0.87; *P* < .00001), reduction in NT-proBNP levels (rate ratio = 0.78; 95% CI, 0.70–0.88; *P* < .0001), and decline in renal function (odds ratio = 0.77; 95% CI, 0.68–0.88; *P* < .0001) compared with ACEis and ARBs. However, there was no statistical difference in the rate of death from cardiovascular causes (HR = 0.86; 95% CI, 0.72–1.03; *P* = .09) between LCZ696 and ACEis and ARBs.

**Conclusion:**

LCZ696 is superior to ACEis and ARBs in the treatment of HF. Hence, it should be more widely used clinically.

## 1. Introduction

Heart failure (HF) is a common multifactorial and complex clinical syndrome characterized by impaired cardiac function. It is the end stage of a chain of cardiovascular events that results in a heavy burden of disease. Thus, it needs early intervention.^[1,2]^ The treatment concept of HF continues to change from the hemodynamic stage in the 1970s to the neuroendocrine stage in the 1990s to the current stage of overall regulation and multi-target action.^[3–5]^ The Golden Triangle drugs include angiotensin-converting enzyme inhibitors (ACEis), angiotensin-receptor blockers (ARBs), and aldosterone receptor antagonists β. They have been recommended by the treatment guidelines as the standard treatment for HF and have changed the treatment landscape for patients with HF in the last 2 decades.^[6,7]^ However, despite treatment advancement, the overall prognosis of HF is poor.^[2]^ With the aging global population, the prevalence of HF continues to increase. The mortality and rehospitalization rates of HF are still high; hence, improvement in treatment modalities should be seriously considered.^[1,2]^ Currently, it is believed that the activation of the neuroendocrine system leading to the myocardial remodeling is the key factor that causes the occurrence and development of HF. The long-term activation of the neuroendocrine system and cytokines is the pathological basis leading to the occurrence and development of HF. Neuroendocrine inhibitors are considered as the cornerstone of the management of HF.^[8,9]^ Nevertheless, the role of traditional neuroendocrine inhibitors in improving exercise tolerance and reducing mortality in patients with HF is generally limited. On the other hand, usage of new neuroendocrine inhibitors is becoming more widely accepted.

LCZ696 is a new neuroendocrine inhibitor that inhibits the occurrence and delays the progression of HF by suppressing myocardial remodeling.^[10,11]^ However, there is a lack of comprehensive comparative study on the efficacy and safety of LCZ696 compared with the traditional neuroendocrine inhibitors, such as ACEis and ARBs, in the treatment of HF. Moreover, whether LCZ696 is superior to ACEis and ARBs remains a matter of great concern. The drug safety can be evaluated in terms of the side effects that the drug confers. The side effects that are associated with neuroendocrine inhibitors include nephrotoxicity, hyperkalemia, symptomatic hypotension, and vascular edema. Among the aforementioned effects, nephrotoxicity is the main index for safety evaluation for drugs.^[12–14]^ Thus, in recent years, several randomized controlled trials (RCTs) investigated the efficacy and safety of LCZ696 in treating HF and compared it with those of ACEis and ARBs. The PARAGON-HF trial^[15]^ reported that LCZ696 did not result in a significantly lower rate of total hospitalizations (rate ratio [RR] = 0.85; 95% confidence interval [CI] 0.72–1.00) compared with ACEis and ARBs among patients with HF and an ejection fraction of 45% or higher. Additionally, the PIONEER-HF trial^[16]^ reported that, although LCZ696 had more advantages than ACEis and ARBs with regards to the reduction of the rehospitalization rate and nephrotoxicity in patients with HF, it had no advantages in controlling the total mortality. On the other hand, other studies yielded contradicting results. The PARADIGM-HF^[17]^ reported that LCZ696 led to a greater reduction (hazard ratio [HR] = 0.84; 95% CI, 0.76–0.93) in all-cause mortality among HF patients compared to that of enalapril. Therefore, the literature is still inconsistent. Thus, whether LCZ696 is more effective and safer than ACEis and ARBs still needs further investigations.

To evaluate the efficacy and safety of LCZ696 in HF, we performed this meta-analysis and examined several important clinical outcomes, including all-cause mortality, rate of hospitalizations, rate of death from cardiovascular causes, change in N-terminal pro-brain natriuretic peptide (NT-proBNP) levels, and decline in renal function. Additionally, we tried to provide clinical evidence of the effects of LCZ696.

## 2. Materials and Methods

### 2.1. Search strategy

We systematically searched the current mainstream medical databases, including PubMed, Embase, and the Cochrane Library, with the inclusive dates being set from inception to February 2022. We covered a vast majority of medical literatures. The search terms used were as follows: “LCZ696,” “sacubitril/valsartan,” “angiotensin receptor neprilysin inhibitor,” “angiotensin-neprilysin inhibitor,” and “heart failure.” We also did a manual search using the reference lists of identified studies to include other potentially eligible literatures. We did not include unpublished papers. When duplicate trials were identified, only the most complete and updated data of the studies were included.

### 2.2. Inclusion and exclusion criteria

The inclusion criteria of the RCTs hereby included in the meta-analyses were as follows: patients diagnosed with HF (population), treatment with LCZ696 (intervention), ACEIs and ARBs (comparison), and one or more outcomes of all-cause mortality, rate of hospitalizations for HF, rate of death from cardiovascular causes, change in NT-proBNP levels, and decline in renal function (outcomes).

The exclusion criteria of were as follows: non-English articles; non-RCTs (reviews, meta-analysis, letters, or case reports), and basic experiments or animal studies.

The trials identified via the search were independently screened for inclusion by 2 authors, namely, C.Y. and H.Q. Any disagreements were arbitrated by a third author, namely, M.D.C.

### 2.3. Data extraction and quality assessment

Two researchers (C.L. and L.J.L.) with knowledge on systematic evaluation independently carried out literature screening and quality evaluation to ensure the objectivity of the process and results. At times of disagreement, a third coauthor (L.R.X.) intervened to reach a final conclusion. The data extracted for each trial were: author, year, trial number, study design, country, number of patients, regimen, and available outcomes for analysis. The HR/RR for the main outcome measures with the relative 95% CI were extracted or calculated from each trial. Two coauthors (L.R.X, H.J.) assessed the methodological quality of selected literatures using the Cochrane Collaboration’s tool.^[18]^

### 2.4. Statistical analysis

We performed the meta-analysis for the extracted data using the Review Manager software (RevMan version 5.4) provided by the Cochrane Collaboration. The data on all-cause mortality, rate of hospitalizations for HF, and rate of death from cardiovascular causes were pooled as HR with a 95% CI, while the data of change in NT-proBNP levels and decline in renal function were pooled as RR and OR with a 95% CI, respectively. The Cochran Q test and I^2^ test were used to assess the heterogeneity between studies. We used a random-effects model for the meta-analysis when the heterogeneity test was statistically significant (*I*^2^ ≥ 50%, *P* < .1). Otherwise, a fixed-effect model was used. A *P*-value of <.05 was considered statistically significant.

## 3. Results

### 3.1. Study selection and characteristics of eligible studies

**Figure 1.**
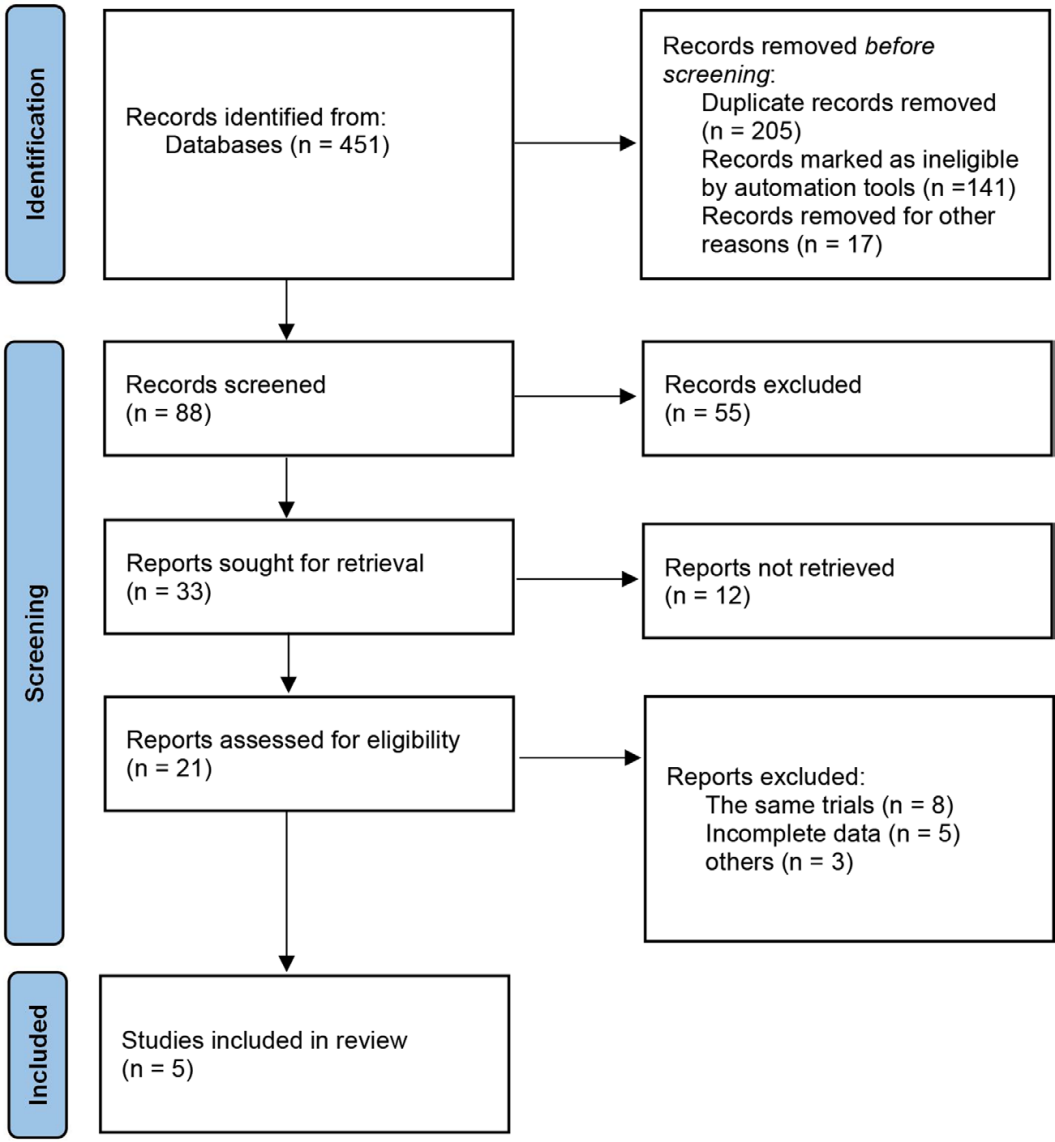
Flow chart about the article selection process.

We identified 451 articles through the databases. After duplicate removal, 5 RCTs^[15–17,19,20]^ involving 19,078 participants were eligible for the meta-analysis (Fig. 1 and Table 1). The number of participants in an individual trial ranged from 301 to 8442. The follow-up time ranged from 8 weeks to 34 months. Among the 5 studies, all patients in the experimental group were diagnosed with HF (4 chronic HF^[15,17,19,20]^ and 1 acute HF^[16]^) and received LCZ696, while those in the control group who were diagnosed with HF received ACEis or ARBs. All articles were published between 2012 and 2021. All 5 studies assessed at least one or more outcomes, namely, all-cause mortality, rate of hospitalizations for HF, rate of death from cardiovascular causes, change in NT-proBNP levels, and decline in renal function. Important details about the included studies are shown in Table 1.

**Table 1:** The main characteristics and outcomes of included studies.

Author/study	Trials number	Yr	Design	Regimen	Country	N	Outcomes used in meta-analysis
Solomon et al^[15]^ (PARAGON-HF)	NCT01920711	2019	Multicenter	LCZ696 (target dose, 97 mg of sacubitril with 103 mg of valsartan twice daily); valsartan (target dose, 160 mg twice daily)	The United States, Britain	4822	Hospitalizations for heart failure Death from cardiovascular causes Renal dysfunction
Velazquez et al^[16]^ (PIONEER-HF)	NCT02554890	2018	Multicenter	LCZ696 (97 mg of sacubitril with 103 mg of valsartan twice daily); enalapril (target dose, 10mg twice daily)	The United States, Britain	881	Mortality Hospitalizations for heart failure Reduction of NT-proBNP Renal dysfunction
McMurray et al^[17]^ (PARADIGM-HF)	NCT01035255	2014	Multicenter	LCZ696 (97 mg of sacubitril with 103mg of valsartan twice daily); enalapril (at a dose of 10 mg twice daily)	The United States, Britain	8442	Mortality Hospitalization for Heart Failure Death from Cardiovascular Causes
Pieske et al^[19]^ (PARALLAX)	NCT03066804	2021	Multicenter	LCZ696 (97 mg of sacubitril with 103mg of valsartan twice daily);e nalapril at a target dose of 10 mg (ACE inhibitors stratum), valsartan at a target dose of 160 mg (ARB stratum)	Multinational	4632	Reduction of NT-proBNP
Solomon et al^[20]^ (PARAMOUNT)	NCT00887588	2012	Multicenter	LCZ696 (97 mg of sacubitril with 103 mg of valsartan twice daily); valsartan (at a dose of 160 mg twice daily)	Multinational	301	Reduction of NT-proBNP Renal dysfunction

NT-proBNP, N-terminal pro-brain natriuretic peptide.

### 3.2. All-cause mortality

**Figure 2.**
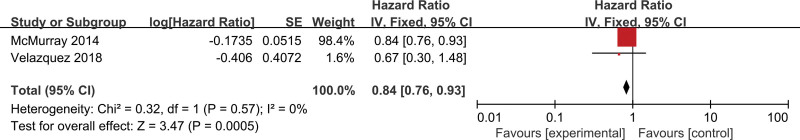
Meta-analysis for the all-cause mortality.

Two studies^[16,17]^ reported the outcome of all-cause mortality, with a total of 9323 patients who received either LCZ696 or ACEis or ARBs. The statistical heterogeneity was deemed low in the pooled effect (*I*^2^ = 0%). There was no significant difference in the mortality rate (HR = 0.84; 95% CI, 0.76–0.93; *P* = .005, Fig. 2) between the 2 groups.

### 3.3. Rate of hospitalizations

**Figure 3.**
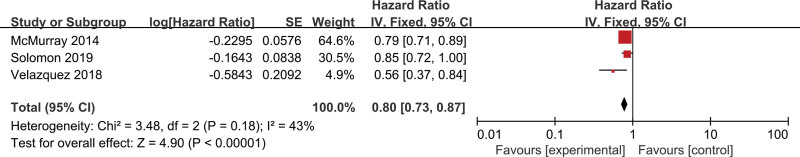
Meta-analysis for the rate of hospitalizations due to HF. HF = heart failure.

Three articles^[15–17]^ examined the hospitalizations for HF, with a total of 14,145 patients. The meta-analysis showed that there was no difference in the rate of hospitalizations between the LCZ696 and the ACEis/ARBs groups (HR = 0.80; 95% CI, 0.73–0.87; *P* < .00001, Fig. 3). Statistical heterogeneity was deemed low across the included studies (*I*^2^ = 43%).

### 3.4. Death from cardiovascular causes

**Figure 4.**
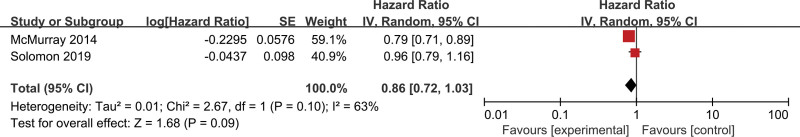
Meta-analysis for death from cardiovascular causes.

Two studies^[15,17]^ with a total of 12,364 patients investigated the changes in death from cardiovascular causes. The statistical heterogeneity was considered high (*I*^2^ = 63%). There was no statistical difference in death from cardiovascular causes between patients who received either LCZ696 or ACEis/ARBs (HR = 0.86; 95% CI, 0.72–1.03; *P* = .09, Fig. 4).

### 3.5. Reduction in NT-proBNP levels

**Figure 5.**
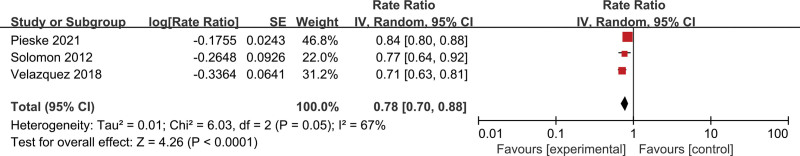
Meta-analysis for change in NT-proBNP level. NT-proBNP = N-terminal pro-brain natriuretic peptide.

Three studies^[16,19,20]^ with a total of 5814 patients reported the changes in NT-proBNP levels. Pooled analysis indicated that administration of LCZ696 resulted in a greater reduction of NT-proBNP level compared with that of ACEis/ARBs (RR = 0.78; 95% CI, 0.70–0.88; *P* < .0001, Fig. 5). High heterogeneity was detected for pooled effect (*I*^2^ = 67%).

### 3.6. Decline in renal function

**Figure 6.**
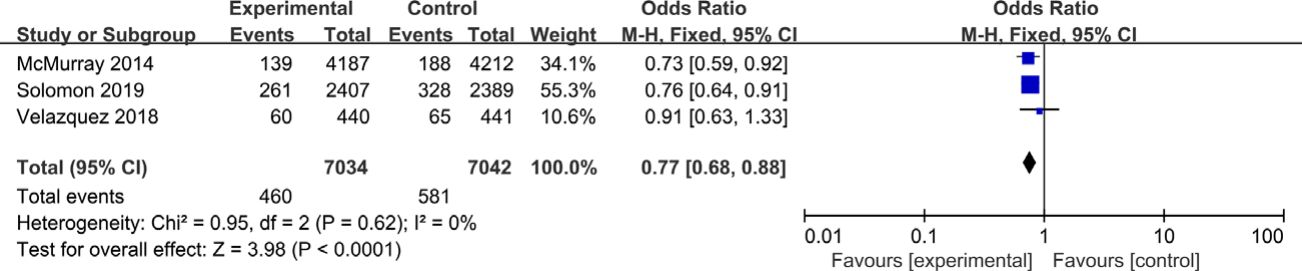
Meta-analysis for decline in renal function.

Three studies^[15,16,20]^ with a total of 14,145 patients investigated the changes in renal function in both the LCZ696 and ACEis/ARBs groups. The analysis indicated that no difference in the decline in renal function was found between the 2 groups (odds ratio = 0.77; 95% CI, 0.68–0.88; *P* < .0001, Fig. 6). The high heterogeneity was statistically significant in all the included studies. No heterogeneity was found among these studies (*I*^2^ = 0%).

### 3.7. Risk of bias assessment

**Figure 7.**
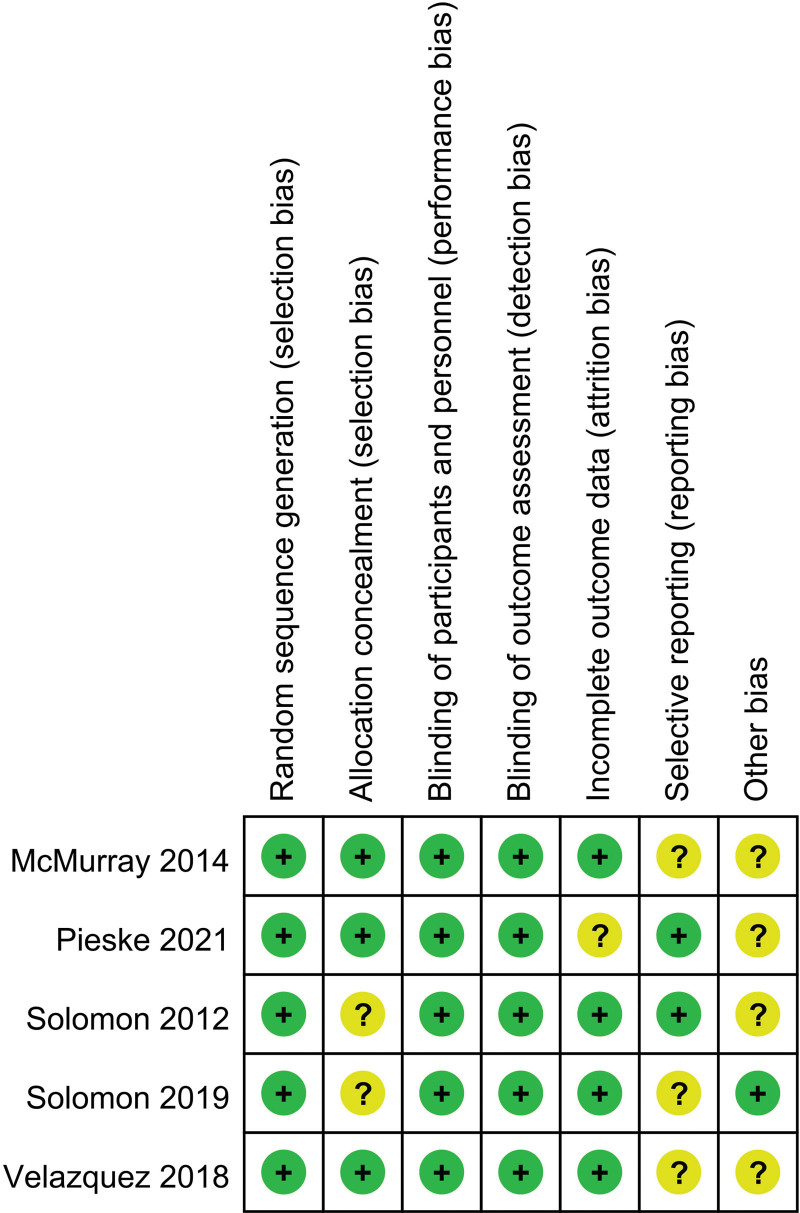
Quality evaluation of included articles.

The risks of bias of the included studies in this meta-analysis are summarized in Figure 7. The methodological quality was assessed as high in all of the 5 included RCTs.

## 4. Discussion

For decades, HF patients have limited effective therapeutic options and a poor prognosis.^[1,3–5]^ The traditional neuroendocrine inhibitors, ACEis and ARBs, inhibit the activity of ACE, reduce the retention of water and sodium, suppresses bradykinin degradation, and relax the blood vessels. It can improve the symptoms and the activity tolerance of patients by reducing the load on the heart, inhibiting the remodeling of myocardial and the sympathetic activity, and protecting the vascular endothelial cells; thus, it reduces the risk of hospitalizations and mortality.^[21]^ However, ACEis and ARBs have adverse reactions, such as nephrotoxicity and hyperkalemia. Particularly, ACEis can cause irritating dry cough. Moreover, it has a poor tolerance.^[22–24]^ By comparison, LCZ696 inhibits ARBs and enkephalinase, which increases the levels of natriuretic peptide, bradykinin, adrenomedullin, and other endogenous vasoactive peptides.^[10,25,26]^ The advent of the angiotensin receptor neprilysin inhibitor, LCZ696, brings new hope for HF patients. However, current literature is not consistent with regards to the superiority of LCZ696 over the traditional drugs, such as ACEis and ARBs. Thus, we performed this meta-analysis to evaluate the efficacy and safety of LCZ696 in HF and sought to find more evidence for the clinical use of LCZ696.

To the best of our knowledge, this is the first meta-analysis that comprehensively compared the efficacy and safety of LCZ696 with those of ACEis and ARBs from various aspects in the treatment of HF. The meta-analysis showed that LCZ696 was superior to ACEis and ARBs in improving the overall mortality, rate of hospitalizations for HF, decline in renal function, and reduction in NT-proBNP levels in patients with HF. These results were consistent with those in Huang et al’s study,^[27]^ in which it was reported that compared with ACEI/ARB, LCZ696 decreased the risk of death, discontinuation due to AEs, and decline in renal function. Moreover, Kang et al^[28]^ reported that LCZ696 significantly increased the estimated glomerular filtration rate (*P* = .02) and reduced the NT-proBNP level (*P* < .001) compared with irbesartan, valsartan, and enalapril. Another study by Chen et al^[29]^ reported that LCZ696 was associated with a significantly reduced risk of renal function deterioration (*P* = .02) compared with that of ACEI/ARB. Furthermore, NT-proBNP is related to the adverse outcomes of HF, and the reduction of NT-proBNP levels and nephrotoxicity are helpful in improving the survival of patients with HF.^[30]^ In addition, evidence shows that renal dysfunction is associated with mortality of HF patients. Moreover, nephrotoxicity is the common adverse reaction of all neuroendocrine inhibitors, which can effectively reduce nephrotoxicity, improve drug tolerance on the one hand, and indirectly reduce mortality.^[8,9,31]^ Therefore, nephrotoxicity is regarded as the primary index to evaluate safety for HF. In this study, we found that, except for the similar rate of death from cardiovascular causes, LCZ696 was superior to ACEis/ARBs in terms of other efficacy and safety outcomes, including the reduction of renal function and reduction of NT-proBNP levels. These findings support the evidence that LCZ696 is superior to ACEis/ARBs in the treatment of HF and is worthy of clinical application.

This meta-analysis has several limitations. First, the number of included studies is relatively small. Nevertheless, the sample sizes of the included studies are very large; hence, the results are quite convincing. Second, due to limited data, we could not perform subgroup analysis according to the types of HF (acute or chronic) and the classification of renal dysfunction; thus, we measured the nephrotoxicity through the increasing blood creatinine ≥ 2 mg/dL and used this as the focus of meta-analysis, which led to a limitation in the evaluation of results in this study. However, we think that the results are more accurate from a larger perspective and supported the conclusion that LCZ696 is superior to ACEis/ARBs in the treatment of HF regardless of the type of HF. Third, angioedema is a common adverse event in patients treated with either LCZ696 or ACEis/ARBs. Since the incidences of angioedema in patients taking LCZ696 and in those taking ACEis/ARBs were reported to be very low (0.2%–0.6% vs 0.2%–1.4%) in the included trials (the PARAGON-HF,^[15]^ PIONEER-HF,^[16]^ and PARADIGM-HF^[17]^ trials), we did not analyze them. However, we think that this toxicity is very important, which requires attention in future researches. Finally, the follow-up time among each trial was different, which may cause a potential impact on the outcome assessments.

## 5. Conclusion

The current meta-analysis demonstrated that, compared with ACEis/ARBs, LCZ696 was associated with a significant improvement in the overall mortality, rate of hospitalizations for HF, reduction in NT-proBNP levels, and decline in renal function for patients with HF. Moreover, it did not increase the risk of death from cardiovascular causes. Due to the limitations in this study, further investigations are required.

## Authors’ contributions

L.Y. and H.Q. contributed to the study design and writing. M.D.C., C.L., and L.J.L. carried out the data collection and selection. L.R.X.and H.J. carried out the data analysis. All authors read and approved the final manuscript.

**Conceptualization:** Yan Chen, Qian He.

**Data curation:** Dun-Chang Mo, Long Chen, Jia-Lu Lu.

**Formal analysis:** Rui-Xing Li, Jie Huang.

**Investigation:** Long Chen.

**Software:** Rui-Xing Li, Jie Huang.

**Writing – review & editing:** Yan Chen, Qian He.
